# Efficacy and safety of glycyrrhizic acid preparation treating comorbid liver injury in COVID-19: A systematic review

**DOI:** 10.3389/fphar.2022.1003697

**Published:** 2022-11-03

**Authors:** Xu Liu, Xia Tian, Zhipeng Ma, Jiali Chen, Qingsong Huang, Peiyang Gao, Chuantao Zhang

**Affiliations:** ^1^ Department of Respiratory Medicine, Hospital of Chengdu University of Traditional Chinese Medicine, Chengdu, China; ^2^ Department of Critical Care Medicine, Hospital of Chengdu University of Traditional Chinese Medicine, Chengdu, China

**Keywords:** COVID-19, SARS-CoV-2, liver injury, glycyrrhizic acid preparation, efficacy, safety, systematic review

## Abstract

**Background:** No specific drug for COVID-19 has been found, and many studies have found that different degrees of liver injury often occurred after infection with COVID-19. Glycyrrhizic acid preparation (GAP) has been frequently used clinically, often combined with conventional treatments such as antiviral therapy, to improve the prognosis of COVID-19 and patients’ liver function.

**Aims:** To critically review and analyze clinical evidence on the efficacy and safety of GAP in the treatment of COVID-19 alone and COVID-19 with comorbid liver injury.

**Methods:** A systematic literature review was performed following a sensitive searching strategy that examines all articles published in “WHO COVID-19 Research Database,” “Cochrane Library,” “VIP,” “CNKI,” “Wanfang,” and “CBM” from 2020 to July 2022. Articles were evaluated by peer reviewers and used Joanna Briggs Institute (JBI) critical appraisal tools to complete the assessment of the risk of bias.

**Results:** Ten clinical studies were finally included, involving 598 patients with COVID-19, of whom 189 were confirmed to be with comorbid liver injury. The main GAPs used are diammonium glycyrrhizinate and magnesium isoglycyrrhizinate, which have shown efficacy in improving liver function, inhibiting inflammation, and enhancing immunity. We are still seeking more related research.

**Conclusion:** Glycyrrhizic acid preparations (mainly diammonium glycyrrhizinate and magnesium isoglycyrrhizinate) have a considerable clinical effect on improving liver function in patients with COVID-19 alone or with comorbid liver injury. Further studies on the use of GAP in the treatment of COVID-19 with comorbid liver injury and its mechanism are still needed.

**Systematic Review Registration**: [www.crd.york.ac.uk/prospero], identifier [CRD42021234647].

## 1 Introduction

An increasing number of studies have noted a close association between COVID-19 and liver injury (Metawea et al., 2021). Elevated liver enzyme levels have been observed in biochemical indicators of COVID-19 patients (Gracia-Ramos et al., 2021; Kim et al., 2021; Yadav et al., 2021; Zhang. et al., 2022), and this elevation is not associated with muscle (Bloom et al., 2021). The mechanisms may include the effects of direct viral cytotoxicity, an exaggerated inflammatory response, ACE2 receptors in the liver or bile ducts, hepatic hypoxia, gut microbiota, vascular endothelial damage, mitochondrial damage, and antiviral drugs (Kariyawasam et al., 2022). Its occurrence at the microscopic level may be mainly related to the distribution of SRB1, TMPRS2, and ACE2 in the liver (Wanner et al., 2022). At present, in COVID-19 patients, the clinical treatment is mainly directed to SARS-CoV-2, using conventional treatments such as antiviral drugs (Wu et al., 2020). There is a lack of attention to the treatment of combined liver injury, and some studies have shown that patients with liver dysfunction such as cirrhosis, fatty liver, and liver transplantation have higher rates of severe illness and mortality after infection with COVID-19 (Ji et al., 2020; Cerbu et al., 2021; Ge et al., 2021; Kim et al., 2021). As for GAP, the animal experiment of Gong et al. (2022) shows that magnesium isoglycyrrhizinate can inhibit bacterial lipopolysaccharide (LPS), Toll-like receptor 4 (TLRs), and nuclear factor-kappa B (NF-κB) signaling pathway to alleviate liver injury induced by anti-tuberculosis drugs. Samsudin et al. (2022) reported that LPS interacts directly with SARS-CoV-2 S protein, which echos the discovery of Gong’s and supports the application of GAP in the treatment of comorbid liver injury in COVID-19. Moreover, the latest research reports that 18β-glycyrrhetinic acid could induce apoptosis in activated hepatic stellate cells to decrease hepatic fibrosis (Zhang et al., 2022b). Thus GAP shows direct and indirect protective effects on the liver. Therefore, this review is dedicated to investigating the clinical efficacy of drugs that can be used for both antiviral and hepatoprotection, GAP. In China and Japan, GAP has been isolated from crude extracts of Gancao and used clinically in the treatment of liver diseases, and has a track record of use in the treatment of SARS-CoV infection (Hoever et al., 2005). Its raw material, Gancao, is one of the most common and frequently used drugs in traditional Chinese medicine (Xiong et al., 2020), specifically the dried roots of *Glycyrrhiza uralensis* Fisch (licorice), *Glycyrrhiza inflata* Bat., *Glycyrrhiza glabra* L. (glycyrrhiza). Gancao’s effects may include anti-arthritic, anti-allergic, anti-cholinergic, anti-estrogenic, anti-inflammatory, anti-leukemic, anti-cancer, inhibition of liver fibrosis (Qu et al., 2012; Tu et al., 2012; Qu et al., 2015), anti-viral (Sun et al., 2019), and anti-hepatotoxic (Cao et al., 2019). It also shows the anti-SARS-CoV-2 activity in the treatment of COVID-19 (van de Sand et al., 2021; Yu et al., 2021), the anti-inflammatory activity of multiple mechanisms (Zhu et al., 2010; Andersson and Tracey 2011; Wang et al., 2011; Mahmoud and Al Dera 2015), the ability to modulate immunity (Gao et al., 2019), and the ability to improve prognosis. It can therefore also be expected to be used to facilitate the treatment and rehabilitation of COVID-19 and COVID-19 with comorbid liver injury.

The efficacy of GAP in COVID-19 has not yet been reviewed by researchers. With a wide variety of GAPs, this study aimed to provide a systematic review of the clinical efficacy and safety of GAP in the treatment of COVID-19 and COVID-19 with comorbid liver injury. The results of this review may provide valuable practical implications about the use of GAP in COVID-19 for patients, healthcare professionals, and those working on COVID-19 research.

## 2 Methods

Our research was conducted according to the Preferred Reporting Items for Systematic Reviews and Meta-Analyses (PRISMA) (Moher et al., 2009), and the protocol has already been published online (Tian et al., 2021). Moreover, given the lack of homogeneity between the studies, we decided to perform a systematic review without a meta-analysis.

### 2.1 Search strategy

All randomized controlled trials, retrospective studies, and clinical trials addressing the use of GAP alone or combined with conventional therapy in the treatment of COVID-19 in Chinese and English were considered for inclusion in our systematic review. Grey literature was also included. We conducted a comprehensive search for articles between 2020 and July 2022 in databases such as WHO COVID-19 Research database (https://search.bvsalud.org/global-literature-on-novel-coronavirus-2019-ncov/), Cochrane Library, VIP, CNKI, Wanfang, and CBM with “glycyrrhi*,” “COVID-19” and similar terms. The detailed search strategy is shown in Supplementary Table S1.

### 2.2 Inclusion criteria

#### 2.2.1 Types of studies

We included randomized controlled trials on GAP for COVID-19 and COVID-19 with comorbid liver injury in the experimental groups. In addition, retrospective studies including cohort studies, case series, and case report, have also been included in this systematic review. The language was limited to English or Chinese.

#### 2.2.2 Types of participants

Patients suffering from COVID-19 alone or with comorbid liver injury were included. Because the population is generally susceptible to SARS-CoV-2, there was no restriction on the age of patients with COVID-19. The confirmation of COVID-19 was when the SARS-CoV-2 was detected by real-time reverse transcription PCR. Liver injury was diagnosed through abnormal liver function tests, which means alanine aminotransaminase (ALT) and/or aspartate aminotransferase (AST) over three times the upper limit unit of normal (ULN); alkaline phosphatase (ALP), gamma-glutamyl transferase (GGT), and/or total bilirubin (TBIL) over two times ULN as liver injury (Cai et al., 2020). When we mention liver injury, we are talking about liver damage occurring due to the virus or its treatment in those without preexisting liver damage. Participants of any sex and ethnicity were all enrolled.

#### 2.2.3 Types of interventions

The experimental group using GAP alone or combined with conventional therapy, and the control group receiving conventional therapy were included. There were no restrictions on the types, dosage forms, doses, and methods of the use of GAP.

#### 2.2.4 Types of outcome measures

##### 2.2.4.1 Primary outcome

Liver function was tested with serum ALT and serum AST. Besides, ALP, GGT, and TBIL were recorded for reference if possible. The disease course will be evaluated by days of hospitalization.

##### 2.2.4.2 Secondary outcome

The mortality rate was defined as the percentage of deaths to the total number after treatment. The blood test was evaluated by C-reactive protein (CRP), white blood cell count (WBC), and procalcitonin. The immune status was assessed by the levels of lymphocytes or CD molecules.

##### 2.2.4.3 Safety outcome

Incidence of adverse reactions was observed by kidney function, bilirubin level, gastrointestinal symptoms (e.g., nausea, vomiting, abdominal pain, diarrhea), rash, and others.

### 2.3 Exclusion criteria

1) Controlled studies in which GAP exists in both the experimental group and the control group; 2) non-clinical research; 3) studies whose intervention was glycyrrhiza extracts (Different from monomer preparations with clear ingredients); 4) patients with preexisting liver disease (e.g., cirrhosis, hepatocellular carcinoma, non-alcoholic fatty liver disease, autoimmune liver diseases, liver transplant or drug-induced liver injury) 5) necessary data unavailable; 6) duplicate studies.

### 2.4 Selection and extraction process

All the literature we retrieved according to prepared keywords and eligibility criteria were all imported into EndNote X9 for classification and sorting, other than duplicate ones. Two researchers (X.L. and Z.M.) independently screened the titles and abstracts of the literature that fulfilled the inclusion criteria. For any potentially related research, we downloaded and read the full text. Discrepancies in the selection processes were resolved by discussion with the third researcher (X.T.). A research flow chart was drawn to show the whole process of research selection.

### 2.5 Data extraction

Two researchers (X.T. and X.L.) independently extracted data and summarized the information from the articles retrieved. Data extraction includes five aspects: 1) basic research information (e.g., title, journal, research ID number, author, contact information, etc.); 2) research methods (e.g., research design, random unit, random method, etc.); 3) observation objects (e.g., age, gender, sample size, etc.); 4) intervention measures (e.g., treatment course information, etc.); 5) measurement indicators (e.g., measurement indicators and time points for judgment, judgment indicators, measurement units, etc.).

We tried to contact the original author when met missing data. Studies with unavailable data were excluded. Similarly, if the data were disputed, it was resolved by discussion. Once the extraction is completed, the two researchers (XT and XL) will check with each other to ensure the accuracy of the data.

### 2.6 Assessment of risk of bias

Each selected paper was assessed individually using Joanna Briggs Institute (JBI) critical appraisal tools (Moola et al.). The appraisal helps to assess the extent to which a study has addressed the possibility of bias and reveals synthesis and interpretation of the results of the study. Two researchers (X.T. and X.L.) were responsible to complete it.

## 3 Results

**FIGURE 1:**
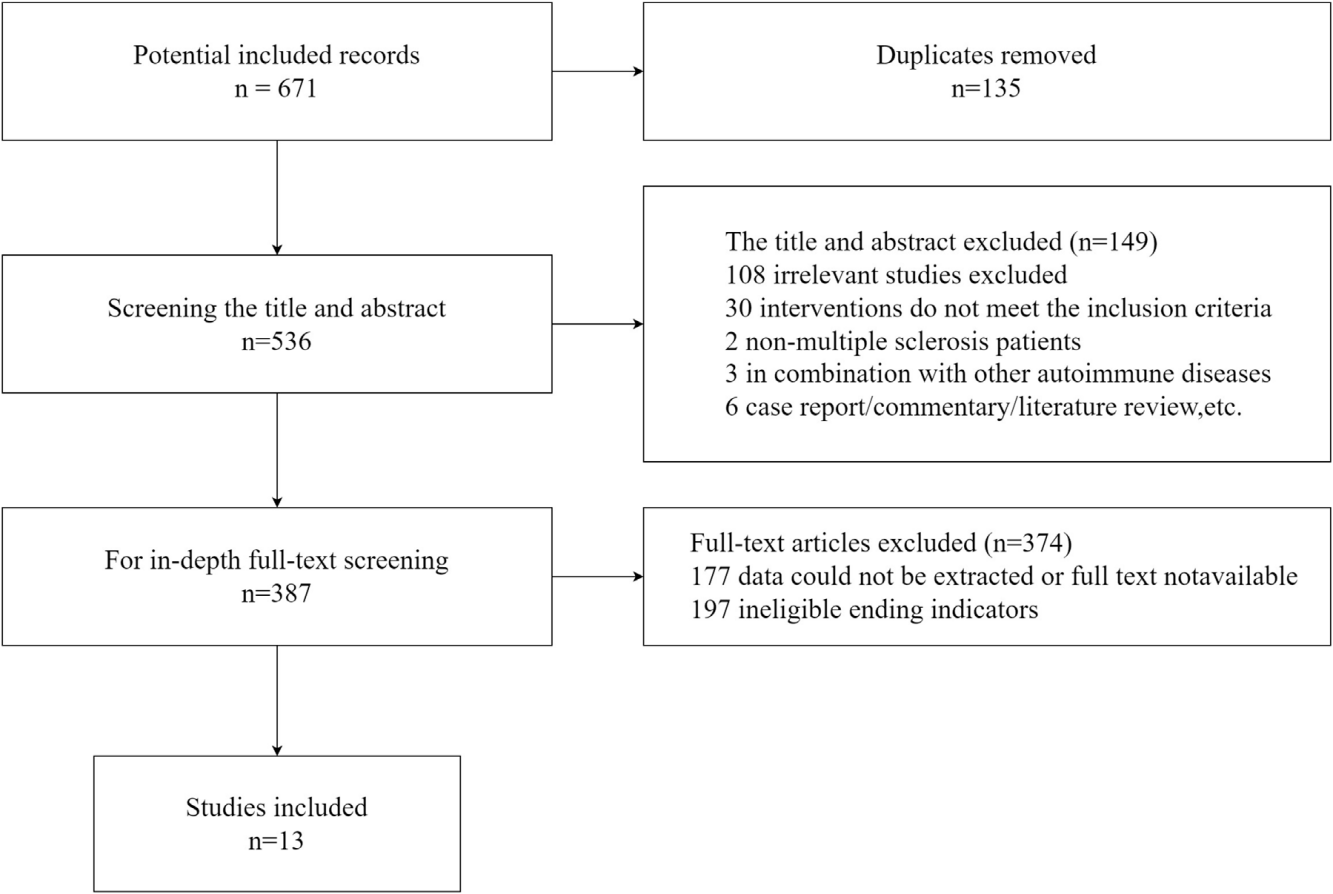
Flow graph of the literature search.

Ten clinical studies, including two randomized controlled trials (Sun et al., 2020; Zhou et al., 2020), four cohort studies (Li et al., 2020; Wang and Lin 2020; Liao et al., 2021; Tan et al., 2022), two case series (Xi et al., 2020; Liu et al., 2021), and two case reports (Liu et al., 2020; He et al., 2022), were ultimately included, of which, four studies observed COVID-19 with comorbid liver injury (Li et al., 2020; Liu et al., 2020; Liao et al., 2021; Liu et al., 2021), while the remaining observed patients with COVID-19 alone. 598 COVID-19 patients were involved, of whom 189 were with comorbid liver injury. In addition, three registered eligible trials were also included (Huo-Shen-Shan Hospital 2020; Pouya Fekran Mahour Pharmaceutical Company 2020; Zhongnan Hospital of Wuhan University 2020). All studies differed in the type of GPA used, study design, and outcomes. The GAPs used in these studies were mainly diammonium glycyrrhizate and magnesium isoglycyrrhizate, and often in combination with antivirals (Li et al., 2020; Sun et al., 2020; Wang and Lin 2020; Xi et al., 2020; Zhou et al., 2020; He et al., 2022; Tan et al., 2022) or vitamin C (Zhongnan Hospital of Wuhan University 2020; Tan et al., 2022). Figure 1 details the flow of screening the studies and the characteristics of the included studies are shown in Supplementary Table S2.

Three studies focusing on COVID-19 alone reported liver function (ALT and AST) (Sun et al., 2020; Wang and Lin 2020; Xi et al., 2020). Xi et al. (2020) reported an increase within the normal range in ALT after the treatment with GAP (*p* < 0.05), which only occurred in 8 out of 46 patients. Sun et al. (2020) and Wang and Lin (2020) found statistically significant differences in both ALT and AST before and after using the GAP. Furthermore, Sun also reported that the ALT and AST levels in the GAP group were lower than in the control group after the intervention. Tan et al. (2022) found that the rate of acute liver injury and the incidence of post-GAP complications, was lower in the group using diammonium glycyrrhizate plus vitamin C than in the control group (arbidol within standard therapy). In addition, Wang and Lin 2020 reported a significant decrease (*p* < 0.05) in both ALP and TBIL after using GAP, and Sun et al. (2020) reported no statistically significant differences in both ALP and GGT. Li et al. (2020) and Liu et al. (2021) found a statistically significant decrease in AST and ALT before and after using the GAP in the treatment of COVID-19 with comorbid liver injury. Liu et al. (2020) reported two patients with COVID-19 treated with magnesium isoglycyrrhizate injection without other antivirals whose mean AST and mean ALT were reduced after the treatment. Liao et al. (2021) temporarily added GAP for 59 patients with COVID-19 to alleviate their abnormal liver function. By the time these patients were discharged or died, AST and ALT in mild and severe cases tended to return to normal levels. And using GAP was more effective in improving ALT and AST levels than not using it in severe cases (Liao et al., 2021). Moreover, Liu et al. (2021) reported a significant decrease in GGT and a slight improvement in TBIL after using the GAP in the treatment of COVID-19 with comorbid liver injury. In contrast, Li et al. (2020) observed that TBIL did not decrease more than in the control group after using GAP in the treatment of COVID-19 with comorbid liver injury and TBIL levels in both groups were within the normal range before and after the intervention.

Three studies suggested that GAP may inhibit the inflammation in the treatment of COVID-19 alone (Wang and Lin 2020; Xi et al., 2020; Zhou et al., 2020), and this effect of GAP is closely related to its shown efficacy in reducing CRP levels. The case series by Xi et al. (2020) reported a statistically insignificant decrease in CRP. The results from Wang and Lin (2020) showed that the use of GAP significantly reduced CRP levels but could not indicate that the group using GAP had a better effect than the control group. The study by Zhou et al. (2020) can support that the use of GAP reduces CRP levels better.

Wang and Lin (2020) observed a statistically significant increase in LYM levels in both GAP and control groups. A similar rise was observed by Xi et al. (2020). Its results were not statistically significant, probably due to insufficient sample size and lack of controls (Xi et al., 2020). The study by Zhou et al. (2020) reported that the use of GAP increased some of the CD molecule levels more than not use. As for WBC, Xi et al. (2020) reported no significant increase, while Tao Wang and Lin (2020) reported a significant increase up to normal level after using GAP, but the difference was not statistically significant when compared with the control group. A study by Li et al. (2020) on COVID-19 with comorbid liver injury showed a significant decrease in CRP after the use of GAP, and the LYM level increased significantly (*p* < 0.05). In addition, the WBC in its experimental group increased but decreased in the control group, and there was no statistically significant difference in WBC between the two groups before and after the treatment (Li et al., 2020). He et al. (2022) reported two cases in which the WBC levels showed a rebounding trend.

In the study by Wang and Lin (2020), the duration of the symptoms including fever, cough, and chest tightness was shorter in the experimental group than that in the control group (*p* < 0.05), and the days of hospitalization were also significantly shorter than that in the control group. The study by Tan et al. (2022) reported that the differences in the days of hospitalization between the observation and control groups were not significant.

A study by Zhou et al. (2020) found that the incidence of adverse reactions (nausea and vomiting, diarrhea, and abnormal liver function) was 13.47% lower in the group using GAP than in the control group, with a statistically significant difference (*p* < 0.05). Xi et al. (2020) not only observed adverse reactions of nausea, but also reported a case of rash, and it resolved on its own without treatment. Tan et al. (2022) reported that the incidence of All new complications [including acute liver injury, acute ciliary muscle injury, acute kidney injury, infectious shock, acute respiratory distress syndrome (ARDS), etc.] in the group using diammonium glycyrrhizate plus vitamin C was significantly lower (19.6% in the experimental group versus 46.1% in the control group), with a statistically significant difference (*p* = 0.00). The incidence of acute liver injury alone was reduced, but the difference was not statistically significant (Tan et al., 2022).

Until 4 July 2022, there were two randomized controlled trials and a cohort study that focus on the treatment of COVID-19 with GAP (Huo-Shen-Shan Hospital 2020; Pouya Fekran Mahour Pharmaceutical Company 2020; Zhongnan Hospital of Wuhan University 2020). They have all registered at the World Health Organization Clinical Trials Registry Platform (WHO ICTRP). The trials researching the effects of GAP on COVID-19 were small in number and size, different in characteristics, and outcome indicators. The sample sizes of these trials were between 60 and 100. Two parallel controlled trials were randomly assigned (Huo-Shen-Shan Hospital 2020; Zhongnan Hospital of Wuhan University 2020). One from Iran used a double-blind method (Pouya Fekran Mahour Pharmaceutical Company 2020) and the other from China was open (Zhongnan Hospital of Wuhan University 2020). In addition, the only single-arm trial was a case series (Huo-Shen-Shan Hospital 2020). The samples of the two trials from China were mainly from hospitals in Wuhan (Huo-Shen-Shan Hospital 2020; Zhongnan Hospital of Wuhan University 2020). All included trials selected different outcome indicators, and the main outcome indicators included measurement of cough severity, the severity of shortness of breath, lung radiologic changes, clinical recovery time, and healing rate, without anyone using liver function indicators.

**FIGURE 2:**
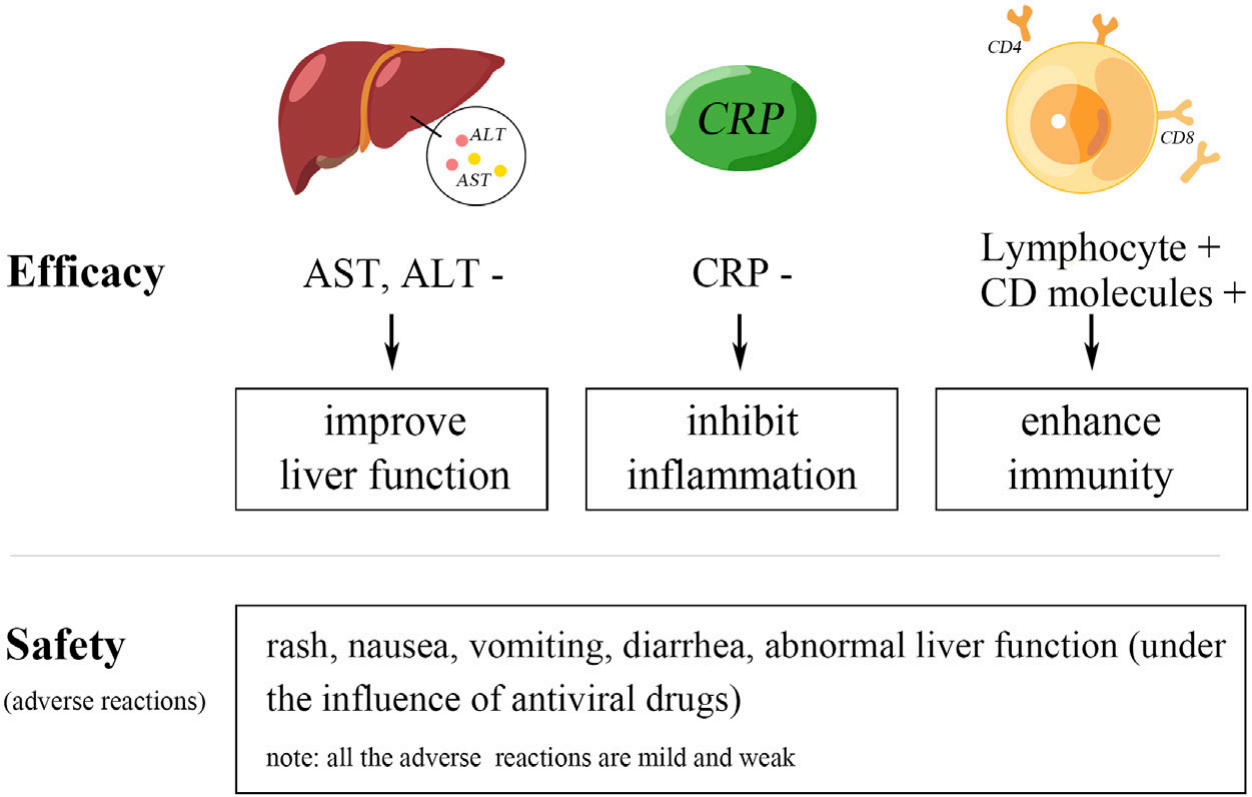
Schematic diagram of the results.

Detailed data is shown longitudinally in Supplementary Table S3 and horizontally in Supplementary Table S4. The efficacy and safety of GAP treating comorbid liver injury in COVID-19 are outlined in the schematic diagram (Figure 2).

All the studies focused on different indicators and varied in quality. The studies by Liu et al. (2020), Liu et al. (2021), and He et al. (2022) had small sample sizes, with less than 10 cases. Moreover, their studies and the study of Xi et al. (2020) all lacked controls (Liu J. et al., 2020; Liu F. et al., 2021; He et al., 2022), which means the results may be not precise enough to reflect the impact of using GAP on COVID-19 patients. However, together with other studies they still have some but limited value, and the different interventions are still a problem. According to the assessment of the JBI’s tool, three of the four cohort studies all failed to analyze or address potential confounding factors (Li et al., 2020; Wang T. and Lin 2020; Liao et al., 2021). Only the study by Tan et al. (2022) used a propensity-score matching (PSM) approach, resulting in less biased results. In the study of Liao et al. (2021), 13 cases were lost to follow-up due to a lack of records, and no further exploration was carried out. The other two randomized controlled trials failed to clearly clarify the allocation concealment and the implementation of blinding (Sun et al., 2020; Zhou et al., 2020). It is not clear whether Sun et al. (2020) used true randomization. These all lead to selection bias (Supplementary Table S5).

## 4 Discussion

We noted that GAP has considerable theoretical advantages and *Gancao* has a long history of use in China and India, not only for treatment but also for disease prevention (Abraham and Florentine 2021). Therefore, we attempt to find clinical evidence to support the use of GAP in the treatment of COVID-19. The evidence we obtained showed that GAP could improve liver function, inhibit inflammation, and enhance the immunity of COVID-19 patients to some extent.

The six studies we included used AST and ALT as outcome indicators. Four experimental groups with n ≤ 24 showed improvements in AST and ALT. In contrast, Xinlin Sun and Jiaxi Xi reported changes within the normal range for groups greater than or equal to 34. In addition, the trends of other liver function indicators differed, thus, we infer that GAP has potential to improve liver function mainly by modulating AST and ALT levels. Further studies and bigger sample size are worth considering. Indicators like CRP, LYM, and CD molecules were researched less than liver enzymes, but also showed similar results, which could partially support the efficacy of GAP in inhibiting inflammation and enhancing immunity.

GAP combined with vitamin C is a common combination. Vitamin C may play a role in antioxidant and scavenging harmful reactive oxygen species (Carr 2020), which can further reduce tissue damage. Liu et al. (2020) found that the AST and ALT levels decreased after using magnesium isoglycyrrhizate injection alone. However, GAP was always used in combination, and some studies used conventional antivirals and supportive therapy when necessary to avoid serious complications and life-threatening events.

GAPs do have some adverse reactions. The studies we included reported rash, nausea, vomiting, diarrhea, and abnormal liver function, but the impact of combining antiviral drugs cannot yet be excluded. Some studies have reported that GAPs may cause adverse reactions such as sodium retention and hypertension (Wu et al., 2018), but in any case, the adverse reactions caused by the use of GAP in the treatment of COVID-19 are few and weak. Washington’s panel reviewed the safety of a series of *Gancao*’s monomer components, including glycyrrhizic acid, ammonium glycyrrhizate, potassium glycyrrhizinate, dipotassium glycyrrhizate, disodium glycyrrhizate, trisodium glycyrrhizate, methyl glycyrrhizate, glycyrrhetinic acid, potassium glycyrrhetinate, disodium succinoyl glycyrrhetinate, glyceryl glycyrrhetinate, glycyrrhetinyl stearate, and stearyl glycyrrhetinate. This study concluded that glycyrrhetinic acid has no acute, short-term, subchronic, or chronic toxicity and that other GAPs can be extrapolated for their safety due to structural similarities (Cosmetic Ingredient Review Expert 2007). The study by Wu et al. (2018) further elucidated the possible adverse reactions of GAPs, noting dose control, control of allergic reactions, contraindications (hypertension, diabetes, heart failure), and that oral formulations have weaker adverse reactions than injectable formulations. In the studies we included, diammonium glycyrrhizate was generally used at 150 mg t.i.d. and magnesium isoglycyrrhizate injection was used at 150 mg q.d. or 100 mg/d. Liao et al. (2021) used only 50 mg diammonium glycyrrhizate t.i.d., temporarily for liver injury.

It was the monomer preparation that we researched, rather than the crude glycyrrhiza extracts. There are still many studies on glycyrrhiza extracts which is more complex in composition. Failure to pay attention to the subtle differences between GAP and glycyrrhiza extracts, or to clearly define the two may lead to biased results (Gomaa and Abdel-Wadood 2021).

### 4.1 Limitation

Since there is no consensus on the target of GAP and its specific mechanism, we cannot define the characteristic indicators of GAP’s efficacy. The number of eligible studies and their scales are also limited. The population involved was mainly middle-aged and elderly people, although the population is generally susceptible to SARS-CoV-2. The wide variation of literature in outcome indicators and study designs made meta-analyses difficult, which reduced the reliability to some extent of our systematic review.

### 4.2 Implication

Liao et al. (2021) found that the combination of GAP did not normalize AST and ALT in critical cases, which may be related to the degree of liver injury, suggesting that future studies need to pay attention to stratifying by the severity of COVID-19. It was not done in some of the studies we included. The study by Xi et al. (2020) did not stratify the type of COVID-19, although the severity of COVID-19 was noted. Moreover, we need more well-designed clinical studies with larger sample sizes to improve the quality of evidence, especially RCTs. Currently, only three registered trials are in progress (Huo-Shen-Shan Hospital 2020; Pouya Fekran Mahour Pharmaceutical Company 2020; Zhongnan Hospital of Wuhan University 2020). When studying the clinical efficacy of GAP on COVID-19, outcome indicators should also consider liver-related indicators, inflammatory indicators, immune indicators, adverse reactions, etc. Researchers should not only analyze the data before and after the treatment with GAP, but also should pay more attention to the differences between the experimental group and control group, and the sources of differences in both of them. Meta-analyses should be implemented when there are more eligible studies emerging. Lastly, elucidating the mechanism of GAP still requires joint efforts of researchers.

This systematic review can support the use of GAP in the treatment of COVID-19 alone or COVID-19 with comorbid liver injury to some extent. In addition, this review has demonstrated again that COVID-19 is prone to comorbiding with different degrees of liver injury. Although mild liver injury in COVID-19 is usually transient and reversible (Cui et al., 2022), monitoring liver function and using hepatoprotective drugs such as GAP is still needed to improve prognosis and enhance the quality of patients’ survival.

## 5 Conclusion

Our article reviewed the studies about the efficacy and safety of GAP on patients with COVID-19 alone or COVID-19 with comorbid liver injury. We found that GAPs (mainly diammonium glycyrrhizinate and magnesium isoglycyrrhizinate) were effective to improve liver function, inhibit inflammation, and strengthen the immunity of patients, with few and mild adverse reactions. These results still need further studies in this field to support and the mechanism of GAPs acting on COVID-19 with comorbid liver injury should be further elucidated.

## Data Availability

The original contributions presented in the study are included in the article/Supplementary Material, further inquiries can be directed to the corresponding authors.

## Supplementary material

Supplementary Table S1 Search strategy.

Supplementary Table S2 Data overview.

Supplementary Table S3 Data longitudinal view.

Supplementary Table S4 Data horizontal view.

Supplementary Table S5 Assessment of risk of bias.

## Abbreviations

COVID-19, coronavirus disease 2019; GAP, glycyrrhizic acid preparation; ALT, alanine aminotransaminase; AST, aspartate aminotransferase; TBIL, total bilirubin; GGT, γ-glutamyl transpeptidase; ALP, alkaline phosphatase; WBC, white blood cell count; LYM, lymphocyte; CD, clusters of differentiation; PCR, polymerase chain reaction
